# Physical Violence and Associated Factors among Housemaids Living in Debre-Tabor Town, Northwest Ethiopia: Does Employer Alcohol Intake Increase Housemaid Violence?

**DOI:** 10.1155/2019/8109898

**Published:** 2019-12-12

**Authors:** Kefyalew Amogne Azanaw, Abebaw Addis Gelagay, Ayenew Molla Lakew, Destaw Fetene Teshome

**Affiliations:** ^1^Department of Nursing, Debre Tabor Health Science College, Ethiopia; ^2^Department of Reproductive and Child Health, College of Medicine and Health Sciences, University of Gondar, Gondar, Ethiopia; ^3^Department of Epidemiology and Biostatistics, College of Medicine and Health Sciences, University of Gondar, Gondar, Ethiopia

## Abstract

**Background:**

Violence against women and girls continues to be a global epidemic, including Ethiopia. Housemaids are a neglected segment of the population, and there are no sufficient findings in our country. This study aimed to assess the magnitude of physical violence and associated factors among housemaids aged 15 years and above living in Debre Tabor town, northwest Ethiopia.

**Methods:**

A community-based cross-sectional study was conducted in Debre Tabor town, northwest Ethiopia from April 1 to 30, 2018. A total of 634 housemaids were selected using cluster sampling method. Data were entered into Epi info version 7.2.2.6 and analyzed with SPSS version 20 using descriptive and analytic statistics. Binary logistic regression analysis was carried out to identify independent significant factors. Adjusted odds ratio (AOR) with 95% confidence interval (CI) was used to report the strength of associations.

**Results:**

From 634 housemaids that participated in the study, 235 (37.1%, 95% CI: 33.1−41.0) of them experienced at least one type of physical violence in their lifetime. Housemaids who previously lived in rural areas (*AOR* = 2.82, 95% CI: 1.61, 4.94), had high working experience (*AOR* = 2.17, 95% CI: 1.27, 3.71), not having parents (*AOR* = 2.02, 95% CI: 1.18, 3.46), being divorced (*AOR* = 2.23, 95% CI: 1.31, 4.20), employer alcohol consumption (*AOR* = 4.97, 95% CI: 2.81, 8.79), and presence of extended family with employers (*AOR* = 2.26, 95% CI: 1.42, 3.59) were independently associated with the probability of housemaid physical violence.

**Conclusion:**

High prevalence of housemaid physical violence has been reported. Socio-demographic characteristics of both employers and employees and the behavioral characteristics of employers contributed to physical violence. It is important to give special attention to housemaids who came from rural areas and do not have parents. It is also important to make work experience of housemaids as short as possible.

## 1. Introduction

According to the World Health Organization (WHO) definition, violence against women is any act of Gender-Based Violence (GBV) that results in, or is likely to result in, physical, sexual, or psychological harm or suffering to women, including threats of such acts, coercion, or arbitrary deprivation of liberty [1]. Universally, all types of violence overlap in a relationship and a significant public health problem, but the level and pattern of violence greatly vary between setting, culture, and segment of the population [2]. Ethiopia is one of the countries where the highest prevalence of both physical and sexual violence against women by an intimate partner is documented [3].

Most studies conducted on violence against women agreed that lower educational status, abuse of alcohol, khat and smoke, and younger women are more likely to increase the experience of violence against women [4–6].

Globally, 10–69 percent of women suffered from physical violence by their intimate partners [7]. In sub-Saharan Africa, the experience of women with physical and/or sexual violence by their intimate partner ranges from 27−59% [8]. In Ethiopia, the prevalence of housemaid physical violence was 16.3% [6].

Gender-based violence is an insidious human right issue and has public consequences [8]. The impact of violence is beyond physical injuries; it also accounts for disability, depression, physical, and reproductive problems, and risky sexual behavior [9].

Worldwide within its informal nature, domestic workers are still challenged to regulation and policy implementation which leads to limited opportunities for access to social and legal protection [10]. Violence often goes unrecognized and unreported, and also masked in a culture of silence. Due to less reportable nature of violence, reliable information on the prevalence of the various forms of violence remains scarce.

Even though there are studies conducted on violence globally and also in the country of Ethiopia, there is a lack of sufficient information about the magnitude and associated factors of physical violence against housemaids. Housemaids are neglected segment of the population, and there is no sufficient information identified as a priority in the national planning documents in Ethiopia. Thus, this study aimed to assess the magnitude and associated factors of physical violence against housemaids.

The finding of the research will provide valuable information to labor and social affairs, health professionals, and policymakers to plan their resource and implementation.

## 2. Materials and Methods

A community-based cross-sectional study was conducted in the Debre Tabor town from April 1 to 30, 2018. The town is located 666 km north of Addis Ababa, the capital city of Ethiopia. All housemaids aged 15 years and above who were living in the Debre Tabor town were considered as the source population. The sample size was calculated using the single population proportion formula assuming 16.3% prevalence of physical violence against housemaids [6], 95% confidence level, and 4% margin of error. Considering a 5% nonresponse rate and using the design effect of 2, the total sample size was 626. Since cluster sampling was employed, 634 individuals were finally sampled.

A cluster sampling technique was employed to select the participants. In the study area, there are four Kebeles (the smallest administrative unit) from which 50% of ketenas (also known as villages) were selected using the simple random sampling technique. The sample size was proportionally allocated for each Kebele, and clusters of households were enumerated from selected ketenas. Then, an eligible participant from each household was selected. All housemaids aged 15 years and above who were living for at least 6 months in the town preceding the data collection period were included.

The dependent variable was physical violence, defined as any acts of slapped or thrown something that could hurt, kicked, pushed or shoved her or pulled her hair, dragged her or beaten her up, hit her with his fist or with something else that could hurt her, choked or burnt her on purpose, and threatened to use or actually used a gun, knife or other weapon against her [11]. Socio-demographic and behavioral characteristics of employers (age, educational status, marital status, religion, occupation, family size, khat chewing, and alcohol drinking) and socio-demographic characteristics of housemaids (age, previous resident, marital status, educational status, religion, family life situation, salary, and working experience as a housemaid in current home) were included as independent variables.

A pretested structured interviewer-administered questionnaire, adopted from the WHO multicountry study, was used to collect data. The questionnaire was written in English and then translated to the local language Amharic. The data were collected by eight trained health extension workers and supervised by four master holder individuals. To ensure data quality, training was given for data collectors and supervisors, and a close follow up and supervision were made.

Data were entered into Epi-info version 7.2.2.6 and exported into SPSS version 20 for analysis. Descriptive analysis was done to describe the data, binary logistic regression analysis was used to identify the associated independent factors. The Adjusted Odds Ratio (AOR) with 95% confidence interval (CI) was used to report the strength of associations of variables. Finally, a significant association was declared at a *p*-value of less than 0.05. The Hosmer and Lemeshow goodness of fit test was also made to check the model fitness. Ethical approval was obtained from the University of Gondar, Institute of Public Health Ethical Review Committee. An official letter was obtained from the Debre Tabor town administration, mayors' office. Verbal informed consent was obtained from participants whose age was 18 years and above. Participants whose age was less than 17 years, verbal assent was obtained from their employers after describing the purpose, benefit, and risk of the study and their right on the decision to participate in the study. Their names were omitted to ensure confidentiality and privacy. The interview was performed at a suitable and secure place. Finally, the questionnaire was cleaned, stored and analyzed at a secured place.

## 3. Results

### 3.1. Socio-Demographic Characteristics of Housemaids

A total of 634 housemaids participated in the study with a 100% response rate. Among the participants, 386 (60.9%) were in the age group of 15−19 years with a median age of 18.5 (IQR: 17, 21) years. Of the total participants, 519 (81.9%) previously lived in a rural area, and 574 (90.5%) were single. Concerning work experience, 491 (71.4%) of housemaids had 1−4 years of work experience, only 251 (39.6%) housemaids had both father and mother, and 141 (22.2%) had no parents (Table 1).

### 3.2. Socio-Demographic Characteristics of Employers

One-fourth, 162 (25.6%), of the employers were in the age group of 30–34 years with a median age of 35 (IQR: 30−40) years. Around half, 325 (51.3%), of the employers had a certificate and above educational level, majority, 538 (84.9%) were married, and 297 (46.8%) were government employees (Table 2).

### 3.3. Prevalence of Physical Violence

**Figure 1:**
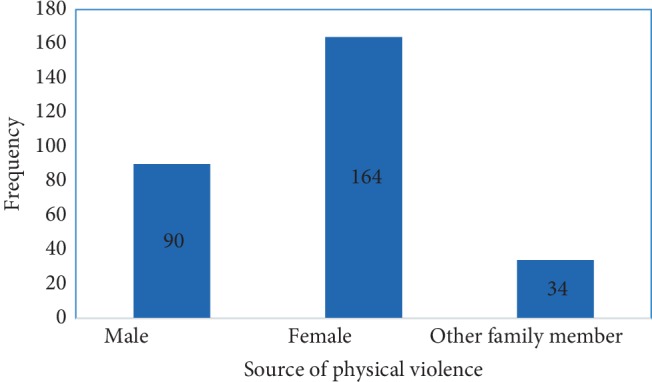
Housemaid physical violence with perpetrators at Debre Tabor town, Ethiopia 2018.

From the participants, 235 (37.1%) (95% CI: 33.1, 41.0) have experienced physical violence during their lifetime while they became housemaids, and 105 (16.6%) (95% CI: 13.7, 19.5) experienced in the last 12 months. For those who experienced lifetime violence, 164 (56.9%) were from their female employers (Figure 1).

### 3.4. Factors Associated with Housemaid Violence

Housemaid's previous resident, working experience, family living situation, employer's marital status, employer alcohol consumption, and extended family living with employers were independently associated with housemaid physical violence.

Accordingly, the odds of physical violence were almost three folds among housemaids who previously lived in rural areas compared to urban (*AOR* = 2.82, 95% CI: 1.61, 4.94). The housemaid who had neither father nor mother was about two times more likely to experience physical violence compared to those who had both father and mother. Either of the male or female employer is an alcohol user increases the likelihood of housemaid physical violence by five folds (*AOR* = 4.97, 95%CI: 2.81, 8.79) (Table 3).

## 4. Discussion

In this study, it is tried to estimate the prevalence of physical violence and identify associated factors among housemaids at the Debre Tabor town, northwest Ethiopia. The prevalence of physical violence during their lifetime, while they became housemaid, was (37.1%) (95% CI: 33.1, 41.0). The finding was consistant with a finding in Bangladesh [12]. However, it is slightly higher compared to a study conducted in the Gondar zuria district, northwest Ethiopia [13], and Germany [14], and significantly higher than a study conducted in the Mekele town, northern Ethiopia [6]. This could partly be due to the difference in the population of interest; the current study included housemaids. Housemaids might not get sufficient information about violence, and they cannot easily protect themselves. Larger prevalence, (43.7%) of physical violence was also reported in the Gozamin district northwest Ethiopia [15].

The odds of physical violence were almost three folds among housemaids who previously lived in rural areas. This finding was consistent with a study conducted in Gondar town northwest Ethiopia [13]. Similar findings have been reported in different areas [16, 17]. The possible reason could be most of the time, housemaids who came from the rural areas might relatively make many mistakes in household activities, and their employers might not be satisfied with their work. Additionally, housemaids who came from rural areas are not empowered to deal effectively with their employers, and they do not know when they could change their employers when their employers attempt to act violently.

In our study, the odds of having physical violence were 2.17 times higher among women with four and above years of working experience as a housemaid. This might be because this study measures a lifetime physical violence, an individual with longer working experience as a housemaid might have a higher chance of experiencing physical violence.

The housemaid who had no or less family support could increase the risk of housemaid violence. In this finding, housemaids who had neither father nor mother or both were about two times more likely to have lifetime physical violence compared to those who had both father and mother. This finding was consistent with another study in northern Ethiopia [6]. The possible reason might be parents' support could increase the confidence in housemaid, and this helps to protect them from any violence. This might also make employers understand that conflict may happen with the parents of the housemaids. Hence, employers may refrain from causing violence.

Divorced or widowed employers increased the odds of housemaid physical violence by a factor of 2.34 compared to married employers. This finding is supported by other studies [12, 18, 19]. This is because divorced or widowed employers might have different problems like psychosocial problems that may lead to envy and depression. A study in 53 countries conducted by the WHO showed that divorced or widowed individuals increased the likelihood of depression [20]. These groups of individuals most likely to be aggressive due to their incompetence in their marriage may be an additional reason.

The odds of physical violence were five times higher among either male or female alcoholic employers. The finding was similar to that of studies conducted in Slovenia, Ghana, Uganda, eastern Sudan, Shimelba refugee camp northern Tigray, Mekele, and Gondar town which revealed that alcohol consumption of employers increased the likelihood of physical violence [5, 6, 13, 19, 21–23]. Many victims of housemaids reported that when the employers took excess alcohol than usual, they assaulted the housemaids. A qualitative study in Debre Tabor town also supported this finding [24]. This is because alcohol has a depressive mental impairment and thus, human beings are encouraged to undertake violence.

An employer having extended family was 2.56 times more likely to increase the probability of housemaids' physical violence. This is because when there are a number of families in the household, the possible sources of violence increased as well, and this increases the likelihood of housemaid violence. Besides, employers with extended family may not have sufficient material assets and income for their expenditure and became more aggressive on their employees.

Though the study did its best to determine the magnitude of physical violence and associated factors in the study setting, it is not free from some limitations. The study was quantitative, and it was better if the qualitative approach was also employed to investigate attitudes of study subjects towards gender-based violence. It may also be exposed to recall bias due to the nature of the cross-sectional design.

## 5. Conclusion

The magnitude of physical violence among housemaids was high. Housemaids coming from rural areas, having more than four years working experience, not having either a mother or a father or both, and employers' alcohol use, having extended family, and being divorced or widowed are the independent factors for housemaid physical violence.

The labor and social affair office and women and children's office have to give special attention and develop strategies to prevent violence of housemaids. It is also important to interrupt the contract of housemaids and employers, and change the behavior of employers so that they do not use alcohol. Further research is needed to address the psychological, sexual, and economic violence of housemaids.

## Figures and Tables

**Table 1:** Socio-demographic characteristics of housemaids, Debre Tabor town, Ethiopia, 2018.

Variables		Frequency (%)
Previous residence	Urban	115 (18.1)
	Rural	519 (81.9)
Educational status	No education	317 (50.0)
	Primary education	181 (28.5)
	Secondary above education	136 (21.5)
Age of housemaid	15–19	386 (60.9)
	20–24	164 (25.9)
	25+	84 (13.2)
Housemaid's religion	Orthodox	588 (92.7)
	Protestant	8 (1.3)
	Muslim	38 (6.0)
Marital status of housemaid	Married	34 (5.4)
	Single	574 (90.5)
	Divorced + widowed	26 (4.1)
Salary of housemaid	100–300	282 (44.5)
	301–500	306 (48.3)
	501+	46 (7.3)
Family living situation	Both alive	251 (39.6)
	Only father alive	112 (17.7)
	Only mother alive	130 (20.5)
	Both dead	141 (22.2)
Work experience	[0.5–2) years	212 (33.4)
	[2–4) years	269 (42.2)
	4+ years	153 (24.1)

**Table 2:** Socio-demographic and behavioral characteristics of employers, Debre Tabor town, Ethiopia 2018.

Variables		Frequency (%)
Age of employer	Below 30	115 (18.1)
	30–34	162 (25.6)
	35–39	112 (17.7)
	40–44	135 (21.3)
	45–49	32 (5.0)
	50 and above	78 (12.3)
Employer's religion	Orthodox	547 (86.3)
	Protestant	22 (3.5)
	Muslim	65 (10.3)
Employer's educational status	Not read and write	53 (8.4)
	Read and write but not formal education	55 (8.7)
	Grade 1–8	63 (9.9)
	Grade 9–12	138 (21.8)
	Certificate and above	325 (51.3)
Employer occupation	Housewife	158 (24.9)
	Government/private employ	297 (46.8)
	Merchant and/driver	168 (26.5)
	Pension	11 (1.7)
Family size	1–3	149 (23.5)
	4–6	425 (67.0)
	7+	60 (9.5)
Marital status of employer	Married	538 (84.9)
	Single	28 (4.4)
	Divorced	37 (5.8)
	Widowed	31 (4.9)
Extended family of employer	Yes	129 (20.3)
	No	505 (79.9)
Khat-chewing	Yes	68 (10.7)
	No	566 (89.3)
Alcohol consumption	Yes	92 (14.5)
	No	542 (85.5)

**Table 3:** Logistic regression analysis to identify factors associated with lifetime physical violence among housemaid, Debre Tabor town, Ethiopia 2018.

Variables		Housemaid violence		*COR* (95% CI)	*AOR* (95% CI)
		Yes	No		
Previous residence	Urban	21	94	1.00	1.00
	Rural	214	305	3.14 (1.89, 5.20)	2.82 (1.61, 4.94)
Work experience	[0.5–2) years	51	161	1.00	1.00
	[2–4) years	99	170	1.84 (1.23, 2.75)	1.35 (0.86, 2.13)
	4+ years	85	68	3.95 (2.52, 6.18)	2.17 (1.27, 3.71)
Age of housemaid in years	15–19	132	254	1.00	
	20–24	71	93	1.47 (1.01, 2.14)	
	25+	32	52	1.18 (0.73, 1.93)	
Salary housemaid	100–300	86	196	1.00	
	301–500	132	174	1.73 (1.23, 2.42)	
	501+	17	29	1.34 (0.69, 2.56)	
Family living situation	Both alive	59	192	1.00	1.00
	Only father alive	53	59	2.92 (1.82, 4.69)	2.02 (1.18, 3.46)
	Only mother alive	60	70	2.79 (1.78, 4.38)	1.97 (1.18, 3.28)
	Both dead	63	78	2.63 (1.69, 4.09)	1.95 (1.20, 3.18)
Family size of employer	1–3	59	90	1.00	
	4–6	161	264	0.93 (0.64, 1.36)	
	7+	15	45	0.51 (0.26, 0.99)	
Marital status of employer	Married	190	348	1.00	1.00
	Single	11	17	1.19 (0.54, 2.58)	1.62 (0.67, 3.91)
	Divorced + widowed	34	34	1.83 (1.10, 3.04)	2.34 (1.31, 4.20)
Extended family	No	164	341	1.00	1.00
	Yes	71	58	2.54 (1.72, 3.77)	2.26 (1.42, 3.59)
Employer alcohol consumption	No	164	378	1.00	1.00
	Yes	71	21	7.79 (4.63, 13.11)	4.97 (2.81, 8.79)

COR: crude odds ratio, AOR: adjusted odds ratio.

## Data Availability

All relevant information is within the manuscript. The data upon which the result was based will be available on request.

## Abbreviations

AOR: Adjusted odds ratio
CI: Confidence interval
COR: Crude odds ratio
GBV: Gender based violence
SPSS: Statistical package for social science
WHO: World health organization.
